# Monitoring Wildlife-Vehicle Collisions in the Information Age: How Smartphones Can Improve Data Collection

**DOI:** 10.1371/journal.pone.0098613

**Published:** 2014-06-04

**Authors:** Daniel D. Olson, John A. Bissonette, Patricia C. Cramer, Ashley D. Green, Scott T. Davis, Patrick J. Jackson, Daniel C. Coster

**Affiliations:** 1 Department of Wildland Resources, Utah State University, Logan, Utah, United States of America; 2 Utah Division of Wildlife Resources, Salt Lake City, Utah, United States of America; 3 Automated Geographic Reference Center, Salt Lake City, Utah, United States of America; 4 Department of Mathematics and Statistics, Utah State University, Logan, Utah, United States of America; The Centre for Research and Technology, Hellas, Greece

## Abstract

**Background:**

Currently there is a critical need for accurate and standardized wildlife-vehicle collision data, because it is the underpinning of mitigation projects that protect both drivers and wildlife. Gathering data can be challenging because wildlife-vehicle collisions occur over broad areas, during all seasons of the year, and in large numbers. Collecting data of this magnitude requires an efficient data collection system. Presently there is no widely adopted system that is both efficient and accurate.

**Methodology/Principal Findings:**

Our objective was to develop and test an integrated smartphone-based system for reporting wildlife-vehicle collision data. The WVC Reporter system we developed consisted of a mobile web application for data collection, a database for centralized storage of data, and a desktop web application for viewing data. The smartphones that we tested for use with the application produced accurate locations (median error = 4.6–5.2 m), and reduced location error 99% versus reporting only the highway/marker. Additionally, mean times for data entry using the mobile web application (22.0–26.5 s) were substantially shorter than using the pen/paper method (52 s). We also found the pen/paper method had a data entry error rate of 10% and those errors were virtually eliminated using the mobile web application. During the first year of use, 6,822 animal carcasses were reported using WVC Reporter. The desktop web application improved access to WVC data and allowed users to easily visualize wildlife-vehicle collision patterns at multiple scales.

**Conclusions/Significance:**

The WVC Reporter integrated several modern technologies into a seamless method for collecting, managing, and using WVC data. As a result, the system increased efficiency in reporting, improved accuracy, and enhanced visualization of data. The development costs for the system were minor relative to the potential benefits of having spatially accurate and temporally current wildlife-vehicle collision data.

## Introduction

Wildlife-Vehicle Collisions (WVCs) are a global problem that impact both wildlife and motorists [1]–[5]. The sheer number of animals that are killed in vehicle-collisions is alarming; in the United States alone it has been estimated that ∼1 million vertebrates are killed every day [6]. Wildlife-vehicle collisions involving large species, such as ungulates, can cause substantial vehicle damage and human injuries, and consequently, are a key public safety concern [7]. In the United States, there are 1–2 million vehicle collisions with large animals each year that result in $8.4 billion (all currency values represent USD) in damages [8]. Additionally, ∼5% of WVCs result in human injuries [7], [8], and in the USA, human fatalities resulting from WVCs have risen to ∼200 annually [9].

There is a current, critical need for accurate and standardized WVC data [10]–[12], because these are the foundation for mitigation projects that protect both motorists and wildlife [13]. For example, exclusionary fencing (>2 m high) is used to prevent wildlife from accessing road right-of-ways, and it is typically only constructed on road sections with high traffic volumes and high numbers of WVCs [14]. Wildlife crossings, which promote connectivity and facilitate safe passage of wildlife above (overpasses, e.g., bridges, green bridges) and below (underpasses, e.g., culverts, tunnels, bridges) roads, are also placed in areas where WVCs occur [15]–[18]. Effective WVC mitigation is generally costly [19], and high quality WVC data help ensure that limited mitigation resources are strategically targeted to areas that produce the greatest results for motorists and wildlife. However, effectively gathering WVC data for mitigation planning has proven challenging [12] because WVCs occur over broad geographic areas, during all seasons of the year, and in large numbers [6], [20]. Collecting data of this magnitude require many observers and an efficient data management system.

Ecologists have been collecting WVC data since at least the 1920s [21]. These early ecologists recorded WVC data manually using the only method available to them at the time: pen and paper. Now almost a century later, many if not most state agencies still use the pen/paper method to report animal carcasses that occur on roadways [12]. This is problematic because data collected in this manner generally have low spatial accuracy (i.e., nearest highway/marker), contain avoidable inaccuracies, and require a considerable time investment to reformat data digitally, so they are useful for analyses and mitigation planning [10]. For instance, data must be entered once on a paper form while in the field and then manually transcribed into an electronic database. After data are in an electronic database, they must then be imported into a Geographic Information System (GIS) to be visually analyzed for mitigation planning. Errors inevitably occur in the process, as humans enter and transcribe WVC data manually, particularly if the handwriting on the paper form is semi-legible. Location data also may be prone to data entry errors. For example, the nearest marker may not be visible from the carcass location or the road may not have any visible markers, making reporting an accurate location difficult or impossible.

Researchers have been aware of the difficulties associated with WVC data for many years, and as a result, have been actively developing new methods with the goal of improving accuracy and efficiency. As early as 2005, Ament et al. [22] developed a system in which observers used Personal Data Assistants (PDAs) to electronically record data on animal carcasses and to generate spatially accurate location coordinates using integrated Global Positioning System (GPS) technology. This system represented a breakthrough in WVC data collection because it not only increased location accuracy, but it also standardized data collection and eliminated transcription errors. Donaldson and Lafon also used this PDA system in Virginia [23]. The use of PDAs, however, did not solve all WVC data collection problems, because PDAs still required the user to periodically transfer data from the PDA to a database for storage, which can be cumbersome when many users are reporting data across large geographic areas. Additionally in about 2006, PDAs began to be replaced by smartphones as the technology of choice. Consequently, PDA reporting systems have not been widely adopted for WVC data collection.

Another reporting system for WVCs was developed by Hesse et al. [24] in 2007. Their system used an inexpensive (∼$100), but lesser known device called the Otto-Driving Companion. This device was attached to the dashboard of the vehicle, and it allowed the motorist to report animal carcasses with the push of a button while driving. The system generated spatially accurate locations using GPS, but was limited by the number species that could be reported. Again, WVC data had to be downloaded manually from each device to a database for the information to be useable. While this represented another step forward in WVC data collection, the Otto-Driving Companion has not been widely adopted.

Most recently, a small number of states and provinces (e.g., California, Idaho, Maine, and British Columbia) have developed web applications for reporting WVCs [25]. These web-based systems allow users to report animal carcasses by accessing a website where they enter location and species information. Some systems even allow users to upload photos of animal carcasses. The development of web applications for reporting WVC data is a significant advancement that standardizes data collection and eliminates transcription, but these systems have two important limitations: 1) users must have internet access, and 2) users must define carcass locations based on what they know about the road location. The requirement of internet access requires personal computer users to either record the data or remember it until they have access to their computer. Some web applications can be accessed with mobile devices, but they require mobile broadband internet which is incomplete in most states, especially in rural areas where many WVCs occur. Web applications also require users to define the locations of WVCs manually, so there is the potential for significant location error to occur. Most web applications now have built-in map viewers (e.g., Google Maps) that allow users to zoom to and select a location on the map, which makes defining the location relatively easy. However, locations errors associated with this technique are unknown and largely dependent on the user.

Presently there is no widely adopted WVC data collection system that is both efficient for users and accurate for geographic locations. Our intent was to create a data collection system that increased efficiency and accuracy, but also had the potential to be broadly accepted and used. We also wanted to create a system that seamlessly integrated WVC data collection, storage, and analysis. In this paper, we review the development and testing of the WVC Reporter. The WVC Reporter is a smartphone-based reporting system that combines a mobile web application for data collection, a centralized database for data storage, and a desktop web application for analyses.

## Methods

### Study Area

The WVC Reporter was developed and tested in Utah (219,807 km^2^), which is located in the southwestern United States. The Utah landscape is topographically diverse with elevations ranging from 663–4,413 m [26]. The climate for much of the state is considered semi-arid (127–381 mm precipitation annually), but high elevation areas can receive considerably more precipitation (>1,473 mm) [27]. Three major ecoregions comprise the majority of the state: the Colorado Plateau, the Wasatch and Uinta Mountains, and the Central Basin and Range [28]. As a result, Utah is ecologically diverse and inhabited by a wide variety of plants and animals that are adapted to an array of habitats from salt desert shrub lands to alpine tundra [29].

Utah is largely a rural state with 75% of the land area being federally or state owned [26]. There are, however, several urban areas along the western front of the Wasatch Mountains in central Utah, where the majority of the state’s 2.8 million residents live [30]. According to the latest census estimate, Utah was the 3^rd^ fastest growing state [31] in the United States. Consequently, the state is rapidly becoming urbanized [32]. The growing human population has increased demand for transportation and traffic volumes have doubled in the past 30 years (1980–2010) [33]. In 2010, it was estimated that 42.8 billion km were driven on the states 73,413 km of roads [33], [34].

Wildlife-vehicle collisions commonly occur in Utah and are a considerable public safety concern [35]. Most reported wildlife vehicle collisions in Utah involve mule deer (*Odocoileus hemionus*) [35], which is the state’s most abundant wild large mammal [36]. Vehicle collisions with mule deer in Utah result in an average of $7.5 million in damages each year [37]. Consequently, mitigation measures such as wildlife crossings and exclusionary fencing have been used to address the problem [38].

### WVC Data Collection

Surveys for wildlife carcasses using automobiles have been conducted systematically in Utah since at least 1998 [39]. Automobile surveys were done by Utah Department of Transportation (UDOT) contractors. During the study, UDOT contractors were contractually obligated to drive ∼2,800 km of roads twice a week (Monday and Thursday) throughout the year. UDOT contractor routes were selected because they had high numbers of WVCs. During surveys, UDOT contractors were required to remove all animal carcasses that were detected on the road surface, the median, and the road shoulder. They also were required to keep detailed records of the species removed and their locations. Removal ensured that carcasses were not double-counted in future surveys, because removed carcasses were transported away from roads by survey crews and deposited at local landfills. The Utah Division of Wildlife Resources (UDWR) employees also reported and removed animal carcasses that occurred on roads other than those covered by UDOT contractors (A. Aoude, UDWR, Pers. Comm.). UDWR employees did not conduct systematic surveys, but reported carcasses opportunistically. Prior to implementation of the WVC Reporter system, both agencies recorded animal carcass data using the pen/paper method.

### WVC Reporter System

**Figure 1:**
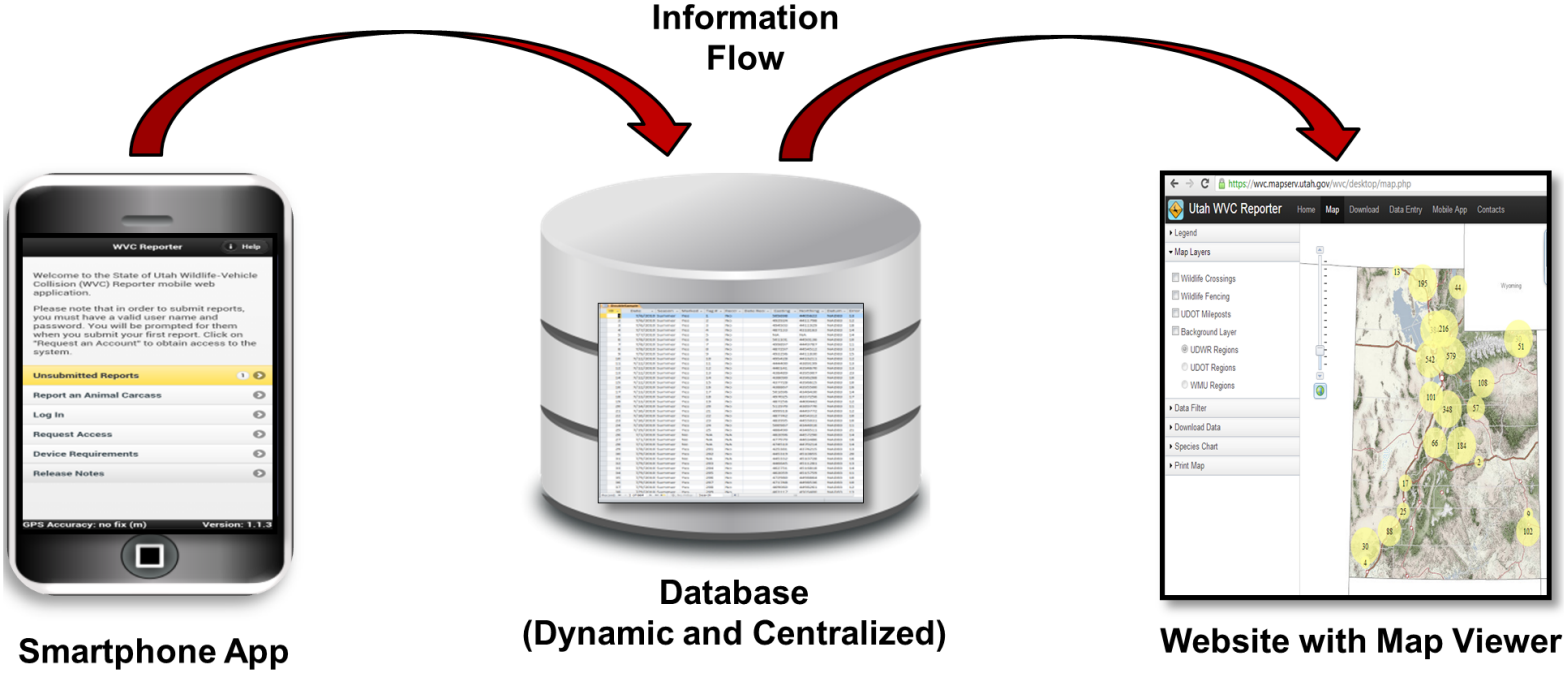
Flow of information through the WVC Reporter system. Using the WVC Reporter system, data are collected in the field using smartphones and a mobile web application. Collected data are then transferred via mobile broadband internet to a centralized database that is dynamically linked to a desktop web application where WVC locations can be viewed.

The WVC Reporter system consists of three integrated components: 1) a mobile web application, 2) a database, and 3) a desktop web application (Figure 1). The mobile web application was designed for in-field data collection. It allows the user to report information on wildlife carcasses using a smartphone. When reporting a wildlife carcass, the user simply clicks on the mobile web application bookmark and a report form opens. The report form contains a dropdown menu of wildlife species that are commonly encountered. If the species being reported is not available in the menu, it can be entered manually. The user also enters the sex (male, female, or unknown) and age class (adult, juvenile, or unknown) of the animal. However, it is important to note that accurately identifying species, sex, age class of animal remains depends on a variety of factors that include observer experience, animal species, and the physical condition of the carcass. Optional information that can be reported with the application includes a carcass fat measurement (an indicator of health in ungulates) and an ID number if the animal was involved in a research study and marked.

For each reported carcass, the mobile application generates a number of pieces of information automatically. For example, the mobile web application accesses the smartphone GPS and acquires coordinates (latitude/longitude) for the location. Coordinates are then used to determine the nearest highway and marker automatically. This eliminates all data entry errors associated with location information. The mobile web application also reports the user, time, and date. When the user is finished entering information in the report form, the send button transfers data via a mobile internet connection to the WVC Reporter database. If mobile internet service is unavailable, the information is stored in the phone cache until the next report is submitted.

The mobile web application is compatible with most iPhone and Android smartphones. Specific device requirements include iOS Safari 3.2+, Android Browser 2.1+, or Google Chrome 10.0+. The programming code for the mobile web application was written in HTML5, CSS, and JavaScript. The HTML5 geolocation Application Program Interface (API) was used to enable location data collection, and the application cache allows the mobile web application to be used even when there is no internet connection available. Programming for all components of the WVC Reporter was done by the Utah Automated Geographic Reference Center (AGRC). The programming code for the system is provided in Appendix S1.

The WVC Reporter database serves as the central repository for all reports that are submitted using the mobile web application. The database is dynamic and updated when reports are submitted through an ESRI ArcGIS Server Feature Service. The database is an ESRI ArcSDE Geodatabase, and it is housed in a Structured Query Language (SQL) Server at the AGRC in Salt Lake City, Utah.

**Figure 2:**
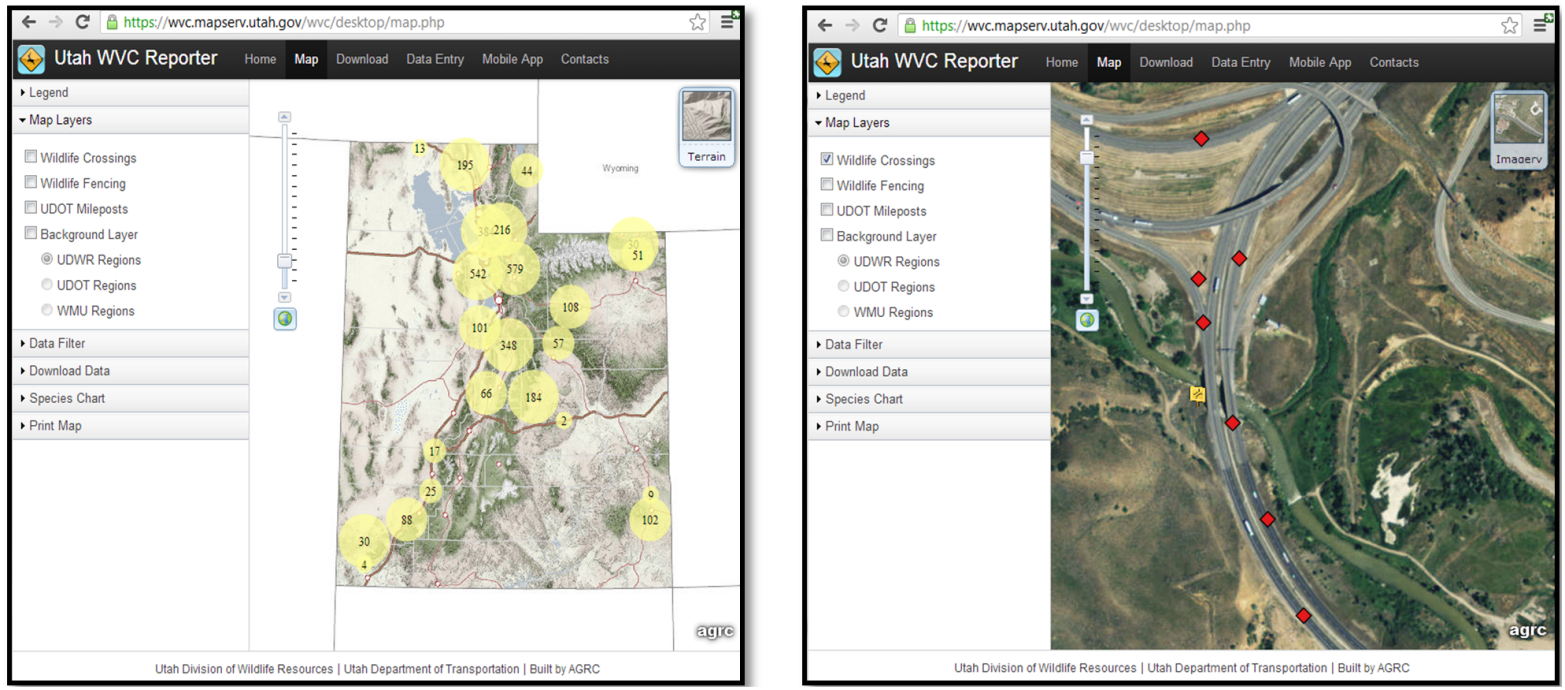
WVC Reporter map viewer depicting spatial patterns in wildlife-vehicle collisions. Spatial patterns in wildlife-vehicle collisions can be efficiently analyzed at both broad (left image) and fine (right image) scale extents using the WVC Reporter map viewer.

The desktop web application was designed to make it easier for planners, maintenance crews, and wildlife managers to use WVC data. To accomplish this, the web application serves as: 1) a map to view carcass locations at user defined scales, 2) a place to download current WVC data, 3) a way to enter carcass data manually, and 4) a link to the mobile web application. To map carcass locations, the desktop web application uses ESRI’s ArcGIS Server and ArcGIS API for JavaScript. The web application is dynamically linked to the WVC Reporter database, so mapped carcass locations represent the most current data available. Rather than display all carcass locations on the map regardless of the spatial extent, the map viewer shows clusters of carcass locations as circles, where the size of the circle represents the number of carcasses in the area (Figure 2). As one zooms in on specific locations within the state, the circles become progressively smaller and eventually disappear at smaller scale extents showing only the actual carcass locations. This provides an efficient means to see where WVC hotspots occur regardless of the scale extent the map is viewed at. Carcass locations also can be overlaid on one of seven different base maps. The high-resolution aerial imagery base map provides an excellent backdrop for analyzing WVC patterns, because landscape features such as vegetation, rivers, human developments, agricultural fields, and roads are clearly visible at smaller scale extents. Additionally, the terrain base map shades relief making topography appear three dimensional, which is helpful for viewing carcass location with respect to major topographic features such as drainages. To add additional context not available in the base maps, we included GIS layers for wildlife crossing locations, exclusionary fencing, marker locations, and management regions (UDOT and UDWR) that can be toggled on and off by the user. The map viewer also includes data filters (date, species, and management region) allowing the user to modify data to suit their specific needs. For fine-scale WVC analysis, users can also enter a highway number (e.g., US 6) and section (e.g., markers 210–213), and the map viewer will zoom to that location and summarize WVC data for that area (Figure 2). Finally, the map viewer allows displayed data to be exported as a PDF, which provides the user with a way to share data or create figures for reports.

While the map viewer provides an efficient means to visualize WVC patterns, in some situations it may be desirable to perform more sophisticated spatial analyses (e.g., spatial clustering or autocorrelation indices). To facilitate this, the desktop web application allows the user to download the WVC Reporter database as either an ESRI shapefile or a dbf file. The shapefile is a common GIS format that allows carcasses location to be easy imported into GIS software where spatial analyses can be performed. The download function also respects the data filters in the desktop web application.

When designing the desktop web application, we realized not all agency personnel reporting WVC collision data would have access to smartphones and consequently some information would still be collected on paper forms. To address this situation, the desktop web application has a report form for manually entering carcass locations. It essentially functions the same as the mobile web application report form, with the exception that the user has to define the carcass location manually by either entering GPS coordinates (latitude/longitude or UTM), the highway/marker, or the street address. Once the location information is entered, the user is able to verify that the location information was correct by viewing the location on a built-in map viewer.

The final function of the desktop web application is to serve as a location to link to the mobile web application. Before field technicians can use the mobile application on their individual smartphones, they must first access the web application (https://wvc.mapserv.utah.gov/wvc/desktop/index.php), click on the mobile app link, and then bookmark the location on their smartphone. The desktop web application was programmed using the same languages as the mobile application, and it works with nearly all commonly used web browsers (Internet Explorer 7+, Chrome, Firefox, and Safari).

### Location Error

We tested the WVC reporter application using a Motorola Droid X smartphone (Model 10083V2-B-K1, Verizon, New York, New York, USA) and an Apple iPhone 4 (Model A1349, Apple, Inc., Cupertino, California, USA). To estimate the horizontal error for locations collected with these phones, we tested them at random locations on highways throughout the state of Utah. At each random location, we recorded location coordinates using a mapping-grade Archer Differential Global Positioning System (DGPS) receiver (Model XF101, Juniper Systems, Logan, Utah, USA) that was capable of sub-meter accuracy. We used locations collected with DGPS receiver to represent the “true” location. Additionally at each random point, we recorded location coordinates using the smartphones and a recreation-grade Garmin GPS receiver (Model eTrex Legend H, Garmin International, Inc., Olathe, Kansas, USA). We included the recreation-grade GPS in testing to determine how the smartphones compared to a standalone GPS receiver. All location data were imported into ArcGIS 10.1 (ESRI, Redlands, California, USA) for analysis. Location error was estimated as the Euclidean distance between the true location and the points collected by the test units. Because the location errors were not normally distributed, we reported the medians and median absolute deviations (MADs) instead of means and standard deviations. We also used the nonparametric Kruskal–Wallis test to test for differences in location errors between units. All statistical tests for this study were performed using R 2.14.1 (R Development Core Team, Vienna, Austria). To estimate how much spatial accuracy improved by using smartphones and WVC Reporter application, we compared location errors associated with that technique to those empirically measured by Gunson et al. [10] for reporting highway/marker locations. We used this information to estimate the percent decrease in location error associated with using smartphones and the WVC Reporter application.

### Data Entry and Transcription Times

We estimated the amount of time required to report carcasses using the WVC Reporter application and the pen/paper method under field conditions. Data entry times can vary based on an individual’s natural ability and experience level. To reduce this bias, all data entry times were collected by the principal investigator, who was experienced entering data using both the pen/paper method and the WVC Reporter application. Data entry and transcription times were recorded in seconds(s) using a stopwatch. For WVC Reporter, data entry times represented the time from when the application was opened on the smartphone until all data was entered and the submit report button was pressed. Data values entered included species, sex, and age class. For the pen/paper method, data entry and transcription times represented the time from when the first and last data values were entered. Values entered included date, highway/marker, species, sex, age class, and GPS coordinates in UTMs. Data entry times were also non-normal, so we reported medians and MADs. We tested for differences in data entry times between methods using the Kruskal–Wallis test.

To determine the how much time could be saved annually, we compared the annual data entry time for the WVC Reporter and the pen/paper method. We estimated annual data entry time for the WVC Reporter by multiplying the median data entry time for each smartphone by the number of carcasses reported during the first year (n = 6,822). Similarly, we calculated annual data entry time for the pen/paper method by multiplying the median data entry by the same number of carcasses (n = 6,822). We then subtracted annual data entry time for the pen/paper from the annual data entry time for the WVC reporter for each phone to get the estimated range of hours saved by using the WVC reporter. A range was reported because the two smartphones tested had slightly different data entry times.

### Data Entry Errors

We estimated reporting errors for the previous system of paper forms and transcription. Data used to estimate entry errors were collected and transcribed by UDOT contractors prior to the implementation of the WVC Reporter system. Due to the nature of the dataset, reporting errors could only be verified for location data. Errors undoubtedly occurred due to misidentification of species, sex, and age information for carcasses, but we did not evaluate these errors because it would have required a separate field study that would have exceeded the financial resources available for this project. Location data collected included highway/marker, and GPS coordinates in UTMs. To identity location errors in carcass records, we imported carcass locations into ArcGIS 10.1 and overlaid them on highway/marker locations to verify that the reported GPS coordinates matched the reported highway/marker locations. If GPS coordinates and highway/marker information matched, we assumed that both had been recorded correctly. When GPS coordinates were associated with a highway, but the reported highway/marker did not match that location, we assumed that the highway/marker was reported incorrectly. When GPS coordinates did not coincide with a highway, we assumed that the coordinates were reported incorrectly.

### Costs Savings

To estimate the total cost savings from using the WVC Reporter, we used the data entry time saved for both in-field data collection and transcription and assumed the mean hourly wage for those reporting and transcribing data was $12/hr.

## Results

### WVC Reporter System

We began development on the WVC Reporter in July of 2011. The system was thoroughly tested for a 6 month period (October 2011–March 2012) prior to its release. Development costs for programming and testing totaled $34,000. Annual maintenance costs were estimated to be $1,500. The WVC Reporter officially went into use across Utah on April 16, 2012. Use of the WVC Reporter application was restricted to UDWR and UDOT personnel, UDOT contractors, and select wildlife and transportation professionals. During the first year of use, 6,822 carcasses were reported by 47 different users across the state. A total of 43 different species were reported, but the majority of carcasses (85%) were mule deer. However, it is important to note that carcass reporting was focused on medium to large mammals because those species posed the greatest threat to driver safety. Smaller species were likely underrepresented because they have lower detection rates and were not a substantial public safety concern.

Spatial patterns were also clearly apparent at multiple scales when using the map viewer to assess carcass locations. For example, the majority of WVCs statewide occurred in the north central portion of the state (Figure 2). At the scale of individual highways, carcasses appeared to be clustered in hotspots along highways. At fine scale resolutions, the landscape and infrastructure features associated with hotspot locations were clearly visible when viewed in conjunction with a high-resolution aerial imagery (Figure 2).

### Location Error

Location error varied between the units we tested (*K* = 25.26, *p*<0.01). The Droid X had the highest median location error (5.2 m). The location error for the iPhone 4 was lower (4.6 m), but similar to the Droid X. The Garmin GPS had the lowest median location error (2.4 m). All units tested produced location data that could be used for precise spatial analysis and mitigation planning. When we compared location errors for data collected with smartphones using the WVC Reporter application to those associated with recording only highway/mile locations (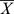 = 401 m, SD = 219 m, reported by Gunson et al. [10]), we found that location error decreased 99% when using the WVC Reporter application. Using a Garmin GPS instead of the smartphones we tested would have further decreased location error <1% (Table 1).

**Table 1:** Horizontal error (m) for locations collected with smartphones (Droid and iPhone) using the WVC reporter and a standalone Garmin GPS receiver.

		Location Error (m)		
Unit	n	Median	MAD	Range
Droid	60	5.2	4.5	0.7–23.2
iPhone	60	4.6	2.9	0.2–21.0
Garmin GPS	60	2.4	1.3	0.3–8.0

Location errors were similar between the smartphones tested, but lower for the Garmin GPS. All units tested produced locations that would allow for precise mitigation planning.

### Data Entry and Transcription Times

Data entry times varied between the methods we tested. (*K* = 225.95, *p* = <0.01). Median entry times using WVC Reporter application (22.0–26.5 s) were 49–58% shorter than the median data entry time (52 s) for the pen/paper method (Table 2). We estimated that the WVC Reporter reduced data entry time by 48.3–56.9 hours per year in Utah.

**Table 2:** A comparison of entry times for data collected with the WVC Reporter application and the pen/paper method.

	Data Entry Time (s)			
Method	n	Median	MAD	Range
WVC Reporter (Droid)	111	22.0	5.9	10.0–42.0
WVC Reporter (iPhone)	122	26.5	9.6	15.0–87.0
Pen/Paper (Garmin GPS)	114	52.0	5.9	41.0–85.0

Data entry times were 49–58% shorter when using the WVC Reporter application.

The median transcription time for observations was 53 s (n = 114, MAD = 3.7, Range = 45–81) As the WVC Reporter completely eliminates manual transcription, we estimated that 100.4 hours were saved per year in Utah on transcription alone. However, it is important to note that transcription times can vary due the ability of the transcriptionist and care with which the original data were recorded.

### Data Entry Errors

We measured data entry error rates for carcasses that were reported using the pen/paper method and then transcribed into an electronic database (Table 3). Data entry error rates were highest for marker locations (19%), intermediate for GPS coordinates (10%), and lowest for highway names (1%). The overall data entry error rate for all location data was 10%.

**Table 3:** Errors for location data that were collected using the pen/method and then transcribed into an electronic spreadsheet.

Location Data	n	Errors	% Error
Highway	1836	23	1.3
Mile Marker	1836	356	19.4
Easting Coordinate	1836	196	10.7
Northing Coordinate	1836	189	10.3
Total	7344	764	10.4

Error rates were highest for mile makers, intermediate for GPS coordinates, and lowest for highway names.

### Cost Savings

Increased efficiency often translates in reduced costs for data collection and use. In Utah, we estimated that 148.7–157.3 hrs of work were saved on entry and transcription of WVC data. As a result, it is possible that $1,784–1,886 in labor costs were saved with the WVC Reporter system, using the assumption that labor costs $12/hr. Additional cost savings almost certainly occurred because data management and analysis were streamlined by the WVC Reporter system, but those savings were not as easy to document and were not estimated in this study.

## Discussion

In 2008, Bissonette and Cramer [11] recommended accurate and standardized WVC data as a priority for transportation planning and wildlife management in North America. Given the recent advances that have taken place in mobile communications and electronics, it seemed promising that WVC data collection could be improved by incorporating these modern advances. The WVC Reporter was specifically designed to leverage modern technologies to produce accurate and standardized WVC data. The system accomplished this by integrating several modern advances (smartphones, GPS, a mobile application, mobile broadband internet, an electronic database, a web application, and a map viewer) into a seamless method for collecting, managing, and using data. The system was developed and tested statewide to serve as a proof of concept, but has the potential to be adopted throughout North America because it produced accurate data, improved efficiency, and enhanced data management and use.

Accuracy was increased by reducing errors associated with location data and by reducing data entry errors. On average, location error for the smartphones we tested was only ∼4–5 m and the largest recorded error for either phone was 23 m. However, location error for highway/marker method can be >800 m, even if locations are reported correctly [10]. Location error of that magnitude can potentially obscure relationships with vegetation, topography, and infrastructure that can be highly variable within an 800 m area. Alternatively, locations collected with smartphones were accurate enough that relationships with landscape features and infrastructure were readily apparent, providing managers with a clearer understanding of factors associated with WVCs at finer spatial scales. Additionally, patterns in WVCs can be influenced by broad scale landscape processes, such as seasonal changes that trigger long distance migrations of ungulates in temperate climates [40], [41]. The seasonal flow of large numbers of migrating ungulates often results in peaks in WVCs in fall and spring [9], [42]. With accurate spatial data on WVCs during migration times, managers will be able to precisely place wildlife crossings at scaled [43] locations were highways insect migration routes, preserving natural ecological processes and reducing vehicle collisions.

With WVC data that is both spatially accurate and temporally current, management can be conducted at a fine scale to address problems as they arise. For instance, deer are occasionally killed on roads that have exclusionary fencing. This can happen when fencing becomes damaged or gates are left open. If maintenance crews observe that deer carcasses are being reported in areas with exclusionary fencing over a short time period of days or weeks, they can examine the location for damaged fencing or open gates, allowing them to quickly address the problem while it is occurring to prevent further WVCs at that location. When WVC data are collected on paper forms, data can be months to years old before they are processed and examined. Subsequently the opportunity to prevent WVCs is reduced. The WVC Reporter also improved data accuracy by reducing errors that occurred from data collection and transcription. When using the pen/method for data collection, ∼10% WVC locations had associated errors. Errors occurred in highway names, marker locations, and GPS coordinates. The highest error rate occurred for marker locations (19%), which was likely due to the fact that markers were not always visible from carcass locations. GPS coordinates, which consist of a long string of numbers (e.g., 12 T 505698 4405622), were also prone to errors (10%) when collected and transcribed manually. Errors in GPS coordinates are especially problematic, because a seemingly innocuous error in which one digit is off by one number can make a location unusable. The errors that occur from manually recording and transcribing data were virtually eliminated using the WVC Reporter because location data were record by the mobile application using the smartphone’s GPS capabilities, rather than by the user manually.

There was also a marked increase in efficiency when we compared the WVC Reporter system to the pen/paper method as data collection time was reduced 49–58% and transcription was eliminated. For one year of reporting in Utah, the time savings from these two factors alone equates to 2.5–4 weeks of work for one person. Time savings could be considerably more for states with higher numbers of WVCs. In one year Pennsylvania had an estimated ∼115,571 deer-vehicle collisions [44]. If we assumed that these data were recorded with the WVC Reporter rather on paper forms, it is possible that 0.8–1.3 person-years of work could be saved. Today state agencies are consistently asked to do more with fewer resources. They may not have the time or person power to process data that requires considerable labor to make it useable for management purposes. The use of WVC Reporter allowed managers to focus on analysis and planning rather than data entry and preparation.

Time savings produced by increased efficiency inevitably translates into reduced costs for agencies. We estimated that in one year $1,784–1,886 were saved in data entry and transcription time in Utah. There are additional savings that occur in data management and analysis. A total of 47 state employees and contractors reported WVC data throughout Utah. Collecting data entry forms from all of those individuals at regular intervals is not trivial; it requires a considerable commitment of time and effort, which is not required with the WVC Reporter system. Additionally data analysis is streamlined with WVC Reporter, because data do not have to be prepared for GIS analysis, and analysis time is reduced because data can be quickly viewed by simply accessing the desktop web application. These cost savings are more difficult to estimate, but are possibly equivalent to or exceed those costs saved on data entry and transcription.

The WVC Reporter had its own associated expenses. System development and testing was moderate ($34,000). Additionally, annual maintenance costs ($1,500) were 4.4% of the development costs. The WVC Reporter system also requires investment in smartphones and wireless data plans. These costs can be partially defrayed by the fact that many people already have smartphones, which would necessitate them only downloading the mobile application at no cost. When WVC Reporter costs are viewed in context of the problem, the investment in the system appears relatively minor. The average economic cost of a deer-vehicle collision has been estimated to be $8,388 and as high as $30,773 for a moose-vehicle collision [8]. Consequently only ∼4 deer-vehicle collisions or ∼1 moose-vehicle collision would need to be prevented to pay for system development. Additionally, if one human fatality could be prevented (estimated value of a human life is $3.3–9.1 million [8], [45], [46]), the system would pay for itself many times over. While Departments of Transportation do not directly bear the majority of expense related to wildlife-vehicle collisions, they are mandated with improving road safety and are motivated to prevent wildlife-vehicle collisions, even though the financial benefits of mitigation (e.g., reduced vehicle repair and injury/fatality costs) due not necessarily return directly to the agency responsible for implementing the mitigation.

While the WVC Reporter has advanced data collection and use, the capabilities of the system could be expanded further. As most smartphones now have built in cameras, the mobile web application could easily be modified to allow users to submit photos of carcasses. Additionally survey effort of users could be quantified by programming the mobile web application to track user’s movements while they are conducting carcass surveys. Quantifying survey effort allows for more rigorous analysis of WVC data. The WVC Reporter system could also be linked to a warning system for drivers. The warning system could be designed as a mobile application that notified drivers whenever they entered an area that was currently experiencing high numbers of WVCs. The alert produced by the warning system could also notify drivers if they are traveling during a time of day when WVCs are more likely to occur (e.g., evening or early morning). This form of warning system would provide drivers with the best information available on WVC conditions. P. W. Johnsen (pers. communication) recently developed the AvoiDeer app for use in Norway (www.avoideer.com) for that purpose. Motorists download the AvoiDeer app and use it to record moose or other animals on the road. Other motorists with the app who approach the location are notified by sound and a visual signal on their phones that roadside wildlife have been sighted at the location. Given the effectiveness of the WVC Reporter in collecting location data, the system could easily be modified to record sightings of live wildlife, to collect data on wildlife crossing infrastructure, or for general maintenance issues like reporting potholes and broken/missing road signs. The applications for this type of technology are broad and could potentially result in significant benefits for agencies, wildlife, and the public.

In just the past 5 years, citizen science has emerged as a powerful tool to address scientific problems that were previously too costly, difficult, or labor intensive for researchers to undertake [47]. Citizen science involves recruiting the general public to collect data for scientific research, and it has the power to focus the efforts of many individuals on large scale problems. WVCs are truly a large scale problem that affects much of the developed world [5], [17], [48]. The scope of the problem is beyond what can be addressed by agencies and researchers alone. For instance in Utah, <4% of the roads were surveyed for carcasses by contractors. Given the ease of data collection and management with the WVC Reporter system, it could easily be extended to a citizen science enterprise where the general public reported WVCs on roads that were not surveyed by agencies. Citizen science programs for WVC data collection have successfully been implemented in California (California Roadkill Observation System [CROS]), Maine (Wildlife Road Watch), and Idaho (Roadkill and Wildlife Salvage) using web applications. The California system (CROS) uses citizens who can print an observation form from the website (http://www.wildlifecrossing.net/california/doc/add_observation), record the information, and then enter the data on the web. Data include type of animal and/or species found, where and when located, how long it might have been dead, pictures of the road-kill, and any additional details about road or traffic conditions. The system then displays a summary of this information for different animal groups across the state. The Maine Audubon Wildlife Road Watch (WRW) also uses citizens to record roadside and road-killed animals (https://maineaudubon.org/wildlife-habitat/wildlife-road-watch/). Observers create an account, and then add observations of species by placing its location on a web-based map. Photos can be uploaded. The Idaho Roadkill and Wildlife Salvage (RWS) does not require citizens to login or register (https://fishandgame.idaho.gov/species/roadkill/add) before reporting sightings of roadkills. The website provides entries for species killed, sex, and a box where observers can check their certainty of identification. This system also provides a web-based map where observers can pinpoint the location of their roadkill observation. Other optional data entries are possible, including whether the observer wants to salvage the animal, a species account box, and time of day as well as observer personal information. Despite the challenges associated with citizen science programs (i.e., inexperienced observers, possible imprecise spatial locations, double reporting, people management), the expansion of WVC data collection to large scales will likely depend on the degree to which the general public can be leveraged using modern electronic reporting systems such as WVC Reporter. In summary, the WVC Reporter is a fully automated system that included a mobile web application for data collection, a database for centralized storage of data, and a desktop web application for viewing data. Because the collection of location data are automated, the only source for error is species ID, sex, and age and those are minor concerns for our system because only trained agency personnel report observations.

## Supporting Information

Appendix S1 **WVC Reporter programming code.**
(ZIP)

Appendix S2 **Location errors.**
(CSV)

Appendix S3 **Data entry times.**
(CSV)

Appendix S4 **Transcription times.**
(CSV)
