# Supplementary material for: Monitoring Wildlife-Vehicle Collisions in the Information Age: How Smartphones Can Improve Data Collection
Source: PLoS One. 2014 Jun 4;9(6):e98613. doi: 10.1371/journal.pone.0098613 (PMC4045807; doi:10.1371/journal.pone.0098613)
Supplement: Appendix S1 — WVC Reporter programming code. (ZIP) [file pone.0098613.s001.zip › WVC Reporter Code/WVC Reporter/desktop/contacts.php]

php include("content/common\_html/common.php") ?


php include("content/common\_html/header.php"); ?

**Scott Davis, Lead Developer**  
Utah AGRC  
stdavis@utah.gov

php include('content/common\_html/footer.php')?
