# Supplementary material for: Monitoring Wildlife-Vehicle Collisions in the Information Age: How Smartphones Can Improve Data Collection
Source: PLoS One. 2014 Jun 4;9(6):e98613. doi: 10.1371/journal.pone.0098613 (PMC4045807; doi:10.1371/journal.pone.0098613)
Supplement: Appendix S1 — WVC Reporter programming code. (ZIP) [file pone.0098613.s001.zip › WVC Reporter Code/WVC Reporter/desktop/content/agrc/widgets/locate/templates/TRSsearch.html]

|  |  |  |  |  |  |
| --- | --- | --- | --- | --- | --- |
| |  |  | | --- | --- | | Meridian | | | SL | UB | | |
| T |  |
| R |  |
| S |  |
| optional | |
| Zoom | | |
