# Supplementary material for: Monitoring Wildlife-Vehicle Collisions in the Information Age: How Smartphones Can Improve Data Collection
Source: PLoS One. 2014 Jun 4;9(6):e98613. doi: 10.1371/journal.pone.0098613 (PMC4045807; doi:10.1371/journal.pone.0098613)
Supplement: Appendix S1 — WVC Reporter programming code. (ZIP) [file pone.0098613.s001.zip › WVC Reporter Code/WVC Reporter/desktop/content/agrc/widgets/locate/templates/ZoomToCoords.html]

Zoom To CoordinatesCoordinate Type:
  

Decimal Degrees
Degrees, Minutes
Degrees, Minutes, Seconds
Universal Transverse Mercator

|  |  |
| --- | --- |
| Longitude (W) |  |
| Latitude (N) |  |

|  |  |  |  |  |  |
| --- | --- | --- | --- | --- | --- |
| Longitude (W) | |  |  |  |  | | --- | --- | --- | --- | |  | ° |  | ' | |
| Latitude (N) | |  |  |  |  | | --- | --- | --- | --- | |  | ° |  | ' | |

|  |  |  |  |  |  |  |  |
| --- | --- | --- | --- | --- | --- | --- | --- |
| Longitude (W) | |  |  |  |  |  |  | | --- | --- | --- | --- | --- | --- | |  | ° |  | ' |  | " | |
| Latitude (N) | |  |  |  |  |  |  | | --- | --- | --- | --- | --- | --- | |  | ° |  | ' |  | " | |

|  |  |
| --- | --- |
| X |  |
| Y |  |

Zoom
