# Supplementary material for: Monitoring Wildlife-Vehicle Collisions in the Information Age: How Smartphones Can Improve Data Collection
Source: PLoS One. 2014 Jun 4;9(6):e98613. doi: 10.1371/journal.pone.0098613 (PMC4045807; doi:10.1371/journal.pone.0098613)
Supplement: Appendix S1 — WVC Reporter programming code. (ZIP) [file pone.0098613.s001.zip › WVC Reporter Code/WVC Reporter/desktop/content/agrc/widgets/tests/BaseMapTests.html]

TEST: BaseMap


Test for the agrc.widgets.map.BaseMap

Defaults  
Add overlay map service with loader shown when drawing.  
Don't use default base map and custom extent.  
Full Extent ButtonBack Button
