# Supplementary material for: Monitoring Wildlife-Vehicle Collisions in the Information Age: How Smartphones Can Improve Data Collection
Source: PLoS One. 2014 Jun 4;9(6):e98613. doi: 10.1371/journal.pone.0098613 (PMC4045807; doi:10.1371/journal.pone.0098613)
Supplement: Appendix S1 — WVC Reporter programming code. (ZIP) [file pone.0098613.s001.zip › WVC Reporter Code/WVC Reporter/desktop/content/html/infoTemplateContent.html]

|  |  |
| --- | --- |
| Gender: | ${GENDER} |
| Age Class: | ${AGE\_CLASS} |
| Xyphoid Measurement (mm): | ${XYPHOID} |
| Collar/Tag#: | ${TAG\_COLLAR\_NUM} |
| Comments: | ${COMMENTS} |
| Road/Highway: | ${HIGHWAY\_ROAD} |
| Address: | ${ADDRESS} |
| Route/Milepost: | ${ROUTE\_MILEPOST} |
| UDOT Region: | ${UDOT\_REGION} |
| UDWR Region: | ${UDWR\_REGION} |
| GPS Accuracy (m): | ${GPS\_ACCURACY} |
| Responder Name: | ${RESPONDER\_NAME} |
| Responder Email: | ${RESPONDER\_EMAIL} |
| Responder Agency: | ${RESPONDER\_AGENCY} |
| WMU: | ${WMU} |
