# Supplementary material for: Monitoring Wildlife-Vehicle Collisions in the Information Age: How Smartphones Can Improve Data Collection
Source: PLoS One. 2014 Jun 4;9(6):e98613. doi: 10.1371/journal.pone.0098613 (PMC4045807; doi:10.1371/journal.pone.0098613)
Supplement: Appendix S1 — WVC Reporter programming code. (ZIP) [file pone.0098613.s001.zip › WVC Reporter Code/WVC Reporter/desktop/content/ijit/widgets/tests/FileUploadTest.htm]

Feedback Test


Maecenas pulvinar volutpat est nec dignissim. Donec volutpat nibh ut lorem convallis
hendrerit. Curabitur lorem odio, imperdiet quis eleifend ac, pellentesque non sem.
Etiam nisl urna, pulvinar eget pharetra sed, condimentum ac felis. Nam mattis lobortis
erat, at bibendum nulla ornare at. In ac tellus mi. Cras vehicula velit in turpis
interdum vel imperdiet leo ultricies. Sed placerat nibh tortor. Vivamus feugiat
odio a nunc laoreet semper. Duis mattis interdum risus at laoreet.

|  |  |
| --- | --- |
| Contract Attachment A | " deleteurl="<%= Url.RouteUrl("project", new {controller="Upload", action="DeleteFile", ProjectNumber="-1"}) %>"> |
| Conflict of Interest Form | " deleteurl="<%= Url.RouteUrl("project", new {controller="Upload", action="DeleteFile", ProjectNumber="-1"}) %>"> |
