# Supplementary material for: Monitoring Wildlife-Vehicle Collisions in the Information Age: How Smartphones Can Improve Data Collection
Source: PLoS One. 2014 Jun 4;9(6):e98613. doi: 10.1371/journal.pone.0098613 (PMC4045807; doi:10.1371/journal.pone.0098613)
Supplement: Appendix S1 — WVC Reporter programming code. (ZIP) [file pone.0098613.s001.zip › WVC Reporter Code/WVC Reporter/desktop/content/ijit/widgets/tests/PageLayoutTests.html]

PageLayout Widget Tests


Note: The BaseMap layout gets offset a bit by the Jasmine test html that's added
after the widget is initiallized. It should work just fine without the tests.
