# Supplementary material for: Monitoring Wildlife-Vehicle Collisions in the Information Age: How Smartphones Can Improve Data Collection
Source: PLoS One. 2014 Jun 4;9(6):e98613. doi: 10.1371/journal.pone.0098613 (PMC4045807; doi:10.1371/journal.pone.0098613)
Supplement: Appendix S1 — WVC Reporter programming code. (ZIP) [file pone.0098613.s001.zip › WVC Reporter Code/WVC Reporter/desktop/dataentry.php]

php include("content/common\_html/common.php") ?


php include ("content/common\_html/header.php");?

Submit Report

Report Date

Species

select a species
or

Gender

- Male
- Female
- Unknown

Age Class

- Juvenile
- Adult
- Unknown

Xyphoid (mm)

Unavailable

Collar/Tag #

Comments

Limited to 255 characters.


Location

- Latitude/Longitude
- UTM
- Route/Milepost
- Street Address

Latitude

Longitude

WGS84

Easting

Northing

Zone 12 NAD83

Route

Milepost

Street Address

Zip Code or City

Verify Location
testing

Submit
 
Clear
testing


php include('content/common\_html/footer.php')?
