# Supplementary material for: Monitoring Wildlife-Vehicle Collisions in the Information Age: How Smartphones Can Improve Data Collection
Source: PLoS One. 2014 Jun 4;9(6):e98613. doi: 10.1371/journal.pone.0098613 (PMC4045807; doi:10.1371/journal.pone.0098613)
Supplement: Appendix S1 — WVC Reporter programming code. (ZIP) [file pone.0098613.s001.zip › WVC Reporter Code/WVC Reporter/desktop/download.php]

php include("content/common\_html/common.php") ?


php include("content/common\_html/header.php"); ?

# Download Data

Data can be downloaded as an ESRI Shapefile or DBF file. You can either download the entire dataset or a subset of the data using the filter below.

- Get the entire database
- Filter for a set of records

## Filter

## Download

php include('content/common\_html/footer.php') ?
