# Supplementary material for: Monitoring Wildlife-Vehicle Collisions in the Information Age: How Smartphones Can Improve Data Collection
Source: PLoS One. 2014 Jun 4;9(6):e98613. doi: 10.1371/journal.pone.0098613 (PMC4045807; doi:10.1371/journal.pone.0098613)
Supplement: Appendix S1 — WVC Reporter programming code. (ZIP) [file pone.0098613.s001.zip › WVC Reporter Code/WVC Reporter/desktop/index.php]

php include("content/common\_html/common.php") ?


php include("content/common\_html/header.php"); ?

## Utah Wildlife-Vehicle Collision Reporter

The Utah Wildlife-Vehicle Collision (WVC) Reporter is a smartphone-based system for reporting animals that have been involved in vehicle collisions. Using this website, animal carcass locations can be viewed on maps that display geographic, highway, and other background information.

View the map »

## Who is collecting the data?

Animal carcass locations are reported by Utah Department of Transportation contactors that that remove carcasses from ~1,200 miles of roads throughout the state. Utah Division of Wildlife Resources employees also report animal carcasses on many highways not covered by Utah Department of Transportation contractors.

## What data is collected?

Animal carcasses are reported by technicians in the field using smartphones and the "Wildlife-Vehicle Collision Reporter Mobile App".

Learn more »

## How is the data used?

Animal carcass data is used in a variety of ways to make highways safer for drivers and wildlife.

Some pages on this website require a user name and password.

Request access »

Website Version: 1.1.1

Release Notes »

php include('content/common\_html/footer.php')?
