# Supplementary material for: Monitoring Wildlife-Vehicle Collisions in the Information Age: How Smartphones Can Improve Data Collection
Source: PLoS One. 2014 Jun 4;9(6):e98613. doi: 10.1371/journal.pone.0098613 (PMC4045807; doi:10.1371/journal.pone.0098613)
Supplement: Appendix S1 — WVC Reporter programming code. (ZIP) [file pone.0098613.s001.zip › WVC Reporter Code/WVC Reporter/desktop/mobileapp.php]

php include("content/common\_html/common.php") ?


php include("content/common\_html/header.php"); ?

# Mobile Web App

Using the smartphone's GPS, this app records the carcass location with an average accuracy of 16 ft. The technician enters information on the species, gender, and age class of the animal. The data is then transmitted to a database where additional information on highway and management region is generated using GIS.

Go to the app »

php include('content/common\_html/footer.php')?
