# Supplementary material for: Monitoring Wildlife-Vehicle Collisions in the Information Age: How Smartphones Can Improve Data Collection
Source: PLoS One. 2014 Jun 4;9(6):e98613. doi: 10.1371/journal.pone.0098613 (PMC4045807; doi:10.1371/journal.pone.0098613)
Supplement: Appendix S1 — WVC Reporter programming code. (ZIP) [file pone.0098613.s001.zip › WVC Reporter Code/WVC Reporter/desktop/release_notes.php]

php include("content/common\_html/stand-alone.php") ?


« Back to Home Page

## Roadkill Reporter Release Notes

| Version | Changes |
| --- | --- |
| 1.1.1 | - Fixed bug with the app displaying the incorrect time for reports. - Fixed a bug preventing mobile users from logging into the desktop map page. |
| 1.1.0 | - Added RESPONDER\_EMAIL, RESPONDER\_AGENCY, RESPONDER\_NAME, & WMU fields to the reports feature class. These fields should now show up in the pop-up on the map as well as the data that is downloaded as either shapefile or .dbf file. - Created script that runs nightly to auto-populate: RESPONDER\_EMAIL, RESPONDER\_AGENCY, RESPONDER\_NAME, ROUTE\_MILEPOST, UDWR\_REGION, UDOT\_REGION, HIGHWAY\_ROAD, & WMU. Please note that these fields will not be populated for new reports until the day after they are submitted to the database. - Added a new filter to the "Data Filter" pane that allows you to filter the data by one or more user email addresses. - Added UDOT Mileposts as a map layer. - Added WMU as a map background layer. - Fixed bug with Chrome freezing when zooming multiple levels at one time. - Moved all calls to https to get rid of unsecured content warnings. - Optimized the filter by Route/Milepost segment server side script. It should work significantly faster now. - Fixed bug with route/milepost filter when using '0' as the from milepost.  \| 1.0.1 \| - Fixed issue with Chrome freezing on the map page. - Fixed issue with the map not displaying all of the points that fall within the selected date range. - Refined log in process. \| \| 1.0.0 \| - Move project to production server and point at wvc.mapserv.utah.gov. - Implemented new UserManagement service. \| \| 0.16.0 \| - Changed name from "Roadkill Reporter" to "Wildlife-Vehicle Collision Reporter" - Fixed bug related to points recorded far from any roads. \| \| 0.15.0 \| - Added Wildlife Fencing layer to map. \| \| 0.14.0 \| - Added ability to enter UTM coordinates on data entry page. - Removed most placeholder text. - Refined pane stack on map page. - Removed Mitigation page. - Added Wildlife Crossings layer. - Added SSL to entire site. \| \| 0.13.0 \| - Added ability to filter by route, from and to mileposts. - Added Species Chart to map sidebar. - Added Print to PDF function to map. \| \| 0.12.0 \| - Data Entry page is now working. You can now manually submit reports. \| \| 0.11.0 \| - Added text and image to the home page. - Added footer to all pages. - Added download and filter functionality to "Download" page. - Fixed flash of instyled content on map page. \| \| 0.10.0 \| - Added Download Data widget to map page. \| \| 0.9.0 \| - Added gender, age class, and region filters. - Fixed some bugs with user authentication. \| \| 0.8.0 \| - Added authentication functionality. \| \| 0.7.0 \| - Fixed point clustering bugs. - Added the ability to filter by one or more species. \| \| 0.6.0 \| - Added loader to map page. - Popup now works better with point clustering. - Re-worked the background layers controls. \| \| 0.5.0 \| - Added release notes page. - Added point clustering to prevent overlaps. - Added the beginnings of the Data Filter widget. You can only filter by report date currently. - Refined popup that displays after clicking on a report. - Loaded data from old database. There are now over 25,000 reports in the database. - Upgraded bootstrap to 1.3.0 and ESRI JSAPI to 2.5 \| |
