# Supplementary material for: Monitoring Wildlife-Vehicle Collisions in the Information Age: How Smartphones Can Improve Data Collection
Source: PLoS One. 2014 Jun 4;9(6):e98613. doi: 10.1371/journal.pone.0098613 (PMC4045807; doi:10.1371/journal.pone.0098613)
Supplement: Appendix S1 — WVC Reporter programming code. (ZIP) [file pone.0098613.s001.zip › WVC Reporter Code/WVC Reporter/mobile/agrc/widgets/tests/ParcelViewerLoaderTests.html]

Parcel Viewer Test Page
