# Supplementary material for: Monitoring Wildlife-Vehicle Collisions in the Information Age: How Smartphones Can Improve Data Collection
Source: PLoS One. 2014 Jun 4;9(6):e98613. doi: 10.1371/journal.pone.0098613 (PMC4045807; doi:10.1371/journal.pone.0098613)
Supplement: Appendix S1 — WVC Reporter programming code. (ZIP) [file pone.0098613.s001.zip › WVC Reporter Code/WVC Reporter/mobile/index.html]

Utah Wildlife-Vehicle Collision Reporter - Mobile


# WVC Reporter

Help

Welcome to the State of Utah Wildlife-Vehicle Collision (WVC) Reporter mobile web application.

Please note that in order to submit reports, you must have a valid user name and password.
You will be prompted for them when you submit your first report.
Click on "Request an Account" to obtain access to the system.

  

- Unsubmitted Reports
- Report an Animal Carcass
- Log In
- Request Access
- Device Requirements
- Release Notes

Log Out

GPS Accuracy: no fix (m)
Version: n/a

Home

# Report

Help

- Species:

  tap to select

  or
- Gender:

  Male

  Female

  Unknown
- Age Class:

  Juvenile

  Adult

  Unknown
- Xyphoid Measurement (mm):


  Unavailable
- Collar/Tag #:
- Comments:
- Cancel

  Submit
- Define Location Manually

  This is required only if your mobile device does not have an integrated GPS receiver.

Home

# Submittal Status

Help

- Unsubmitted Reports
- Report Another Carcass

Home

# Reports Queue

Help

You have   report(s) stored on your device that need to be submitted to the server.

Submit Reports

Home

# Release Notes

Help

1.1.3  

- Attempted to address a rare bug that causes the report location to be undefined.

1.1.2  

- Temporarily fixed geolocation bug related to iOS 6. Please remove any home page links and re-add them to complete the fix.

1.1.1  

- Fixed a rare bug related to defining location manually.

1.1.0  

- Added nightly script to update nearest road, regions and milepost data. This allowed for the removal of code in the mobile app that previously calculated these values. This significantly simplifies the code for submitting a new report which should hopefully result in better performance and fewer errors.
- Removed prompt to log in when a report is submitted.
- Other miscellaneous bug fixes and improvements.

1.0.2  

- Refined log in process.
- Upgraded jQueryMobile to 1.1.
- Minor bug fixes.

1.0.0  

- Pointed at ArcGIS\_Roadkill instance.
- Pointed at new UserManagementService.

0.16.0  

- Fixed bug with locally stored reports not successfully submitting.
- Added "Initialize Debug Session" button to help page.

0.15.0  

- Added "Submit Diagnostics to Support" button to the help page.
- Added more error reporting.

0.14.0  

- Changed name from "Roadkill Reporter" to "Wildlife-Vehicle Collision Reporter"
- Added "Define Location Manually" button.
- Reduced maximum buffer distance for nearest road search.
- Fixed bug related to points recorded far from any roads.

0.13.0  

- Added error reporting capabilities on the back end to enable better bug tracking.

0.12.0  

- Fixed bug with logging in.

0.11.0  

- Wired up new user registration page.
- Fully implemented authentication. To get access to submit reports you will need to have a validate user account.

0.10.0  

- Upgraded to jQuery Mobile release candidate 1
- Added prompt to Submittal Status page to prompt users to submit offline reports, if any.

0.9.0  

- Added mock login/out pages.
- Fixed date submitted bug.
- Enhanced form layout.

0.8.1  

- Upgraded to jQuery Mobile beta 3.

0.8.0  

- Xyphoid measurement is now set to unavailable as default.
- Xyphoid measurement stores a "-999" if the unavailable checkbox is checked.
- The form is now validated to make sure that all required values are present before submission.
- Added more emphasis when there are locally stored reports that need to be submitted.

0.7.0  

- The road/hwy is now populated automatically when the form is loaded.
- Added Help page.
- Added green and yellow background colors to indicate submission status on the confirmation page.
- Added a dialog that appears if there is no gps fix available on form submit that asks for lat/long, route/milepost, or an address.

0.6.0  

- Updated species list and added the text box for typing in a new species.
- Added functionallity to auto-detect the road.
- Increased xyphoid fat max to 40 and added the "mm".
- Added support for offline. Reports can now be stored locally on the device for later submission to the server.
- Added prompt to add to home screen on iOS devices.

Back

# Help

#### Reports

If you have a mobile device with an integrated GPS receiver, your location will be automatically recorded when you click on the "Submit" button.
If you have a laptop you can click on the "Define Location Manually" button to record your location.

Please complete one report per animal instead of including another animal in the comments section.

#### When operating the application offline

Leave the Hwy/Road field blank to have the system auto-detect it when you later submit the reports.

Please **do not clear your internet cache**. If you do, then any offline reports will be lost.

#### How to add a shortcut to your home screen on an Android device

Open the application in your web browser. Tap Menu → Add bookmark → Add. Then add it to your home screen by tapping Menu → Bookmarks → long-tap your newly created bookmark → Add shortcut to Home. Because of differences between Android device manufacturers your device may be slightly different.

---

You can send an email to support if you have any questions or problems.

---


Send Diagnostics to Tech Support
Initialize Debug Session

There is no gps fix available. Please manually input your location information below.

Latitude:

Longitude:


or

Route:

Milepost:


or

Street Address:

Zip Code or City:


Cancel

Submit

Home

# Device Requirements

Help

## Supported Browsers

Browser | Min Version |
| Android Browser | 2.1 |
| iOS Safari | 3.2 |
| Chrome | 10.0 |

- Email
- Password
 

Cancel

Log In

Home

# Request Access

Help

- First Name
- Last Name
- Agency or Business Name
- Email
- Verify Email
- Password
- Verify Password

Passwords must be at least 7 characters in length and include at least 1 non-alphanumeric character.

Submit

You have some offline reports that need to be submitted. Would you like to submit them now?

No

Yes

OK
